# Preterm Infants Fed Cow's Milk-Derived Fortifier Had Adverse Outcomes Despite a Base Diet of Only Mother's Own Milk

**DOI:** 10.1089/bfm.2019.0133

**Published:** 2020-05-08

**Authors:** Alan Lucas, John Boscardin, Steven A. Abrams

**Affiliations:** ^1^ Institute of Child Health, University College, London, United Kingdom.; ^2^ Department of Medicine and University of California, San Francisco, California, USA.; ^3^ Department of Epidemiology & Biostatistics, University of California, San Francisco, California, USA.; ^4^ Department of Pediatrics, Dell Medical School, The University of Texas, Austin, Texas, USA.

**Keywords:** human milk, infant feeding, human nutrition, fortifiers, human milk-derived fortifier, cow's milk-derived fortifier, necrotising enterocolitis

## Abstract

***Objective:*** An increasingly common practice is to feed preterm infants a base diet comprising only human milk (HM), usually fortified with a cow's milk (CM)-derived fortifier (CMDF). We evaluated the safety of CMDF in a diet of 100% mother's own milk (MOM) against a HM-derived fortifier (HMDF). To date, this has received little research attention.

***Study Design:*** We reanalyzed a 12-center randomized trial, originally comparing exclusive HM feeding, including MOM, donor milk (DM), and HMDF, versus a CM exposed group fed MOM, preterm formula (PTF), and CMDF1. However, for the current study, we performed a subgroup analysis (*n* = 114) selecting only infants receiving 100% MOM base diet plus fortification, and fed no DM or PTF. This allowed for an isolated comparison of fortifier type: CMDF versus HMDF to evaluate the primary outcomes: necrotizing enterocolitis (NEC) and a severe morbidity index of NEC surgery or death; and several secondary outcomes.

***Results:*** CMDF and HMDF groups had similar baseline characteristics. CMDF was associated with higher risk of NEC; relative risk (RR) 4.2 (*p* = 0.038), NEC surgery or death (RR 5.1, *p* = 0.014); and reduced head circumference gain (*p* = 0.04).

***Conclusions:*** In neonates fed, as currently recommended with a MOM-based diet, the safety of CMDF when compared to HMDF has been little researched. We conclude that available evidence points to an increase in adverse outcomes with CMDF, including NEC and severe morbidity comprising NEC surgery or death.

## Introduction

Evidence indicates human milk (HM) feeding in preterm infants, especially in those under 1,500 g (very low birth weight [VLBW]), has a lower risk of adverse outcomes compared to feeding these infants wholly or partly cow's milk (CM)-based products in terms of necrotizing enterocolitis (NEC),^1–4^ late onset sepsis,^5–8^ mortality,^7,8^ retinopathy of prematurity (ROP),^7,9–11^ bronchopulmonary dysplasia (BPD),^7,10,12^ and long-term brain development,^13,14^ cardiovascular risk,^15–17^ bone health,^18^ atopic disease^19^ and structural development of the heart, lungs, and great vessels.^20^ The extent to which these outcomes reflect beneficial effects of HM or conversely adverse effects of CM (or both) is unknown, but regardless of mechanism, such data underpin the strong recommendation to use mother's own milk (MOM) for VLBW infants.^21,22^

When MOM is insufficient, a preterm formula (PTF) has been used. However, the current recommendation of the American Academy of Pediatrics and other groups^21,22^ is that VLBW infants should, in that case, ideally receive donor milk (DM) rather than PTF providing a 100% HM base diet. This approach requires fortification of the HM to meet nutritional needs of VLBW infants. While many units elect to use HM-derived fortifier (HMDF) as a method of fortification, most currently use of a CM-derived fortifier (CMDF). This practice involves a greater use of HM, the elimination of PTF, but a greater use of CMDF. Yet, the safety aspects of CMDF, in terms of whether there is an increased risk of morbidities as noted above, have been little researched in relationship to this particular feeding guideline.

To address the paucity of evidence on morbidity with this emerging practice, we reanalyzed data from a 12-center clinical trial published in 2010.^1^ This trial originally compared HM feeding (MOM plus DM, if required, and both fortified with HMDF) to a control group fed MOM fortified with CMDF plus use of preterm infant formula when MOM was insufficient. To replicate the commonly recommended practice, we conducted a subgroup analysis confined to babies whose base diet was 100% MOM and a fortifier was supplemental to this, allowing comparison of CMDF versus HMDF in otherwise entirely MOM-fed babies. Our primary hypothesis was that when used in this situation, CMDF would adversely impact the incidence of NEC, NEC surgery and/or death.

## Materials and Methods

### Relevant aspects of the original trial

Infants were recruited from 12 neonatal units, 11 in the United States and one in Austria. Eligibility criteria included birth weight between 500 and 1,250 g, mother's intention to provide her milk, and initiation of enteral feeding before 21 days and parenteral nutrition within 48 hours after birth. Exclusions included major malformations or likely transfer to nonstudy institutions. This trial used block, stratified (by birthweight and small for gestational age [SGA] status) randomization and described previously.^1^ The study was approved by the Institutional Review Boards of each center, and written informed consent was obtained from the parents or legal guardians of all subjects before enrollment. The original trial was registered with Clinicaltrials.gov reg. #NCT00506584.

All study infants received MOM and were randomized to fortifier type received and type of milk used if MOM was insufficient. Two exclusively HM-fed groups, HM100 and HM40, received pasteurized donor HMDF (Prolact+H2MF; Prolacta Bioscience, City of Industry, California) when the enteral intake was 100 and 40 mL/[kg·day], respectively, and both groups received pasteurized and standardized 20 kcal/oz donor HM if MOM was not available in sufficient quantity. The CM control group received CMDF when the enteral intake was 100 mL/[kg·day] and PTF if MOM was not available in sufficient quantity.

Study duration was the earliest of the following: 91 days of age, discharge from hospital, or attainment of 50% oral feedings. Trophic feedings were initiated 1–4 days postnatally and continued at 10–20 mL/[kg·day] as tolerated for up to 5 days. Subsequently, milk intake was increased by 10–20 mL/[kg·day]. HMDF was added at 40 or 100 mL/[kg·day]; the HM40 group tested a further hypothesis that HMDF could safely be used earlier than the conventional time of introduction of a fortifier. CMDF (Enfamil HMF; Mead Johnson, Evansville, IN; or Similac HMF; Abbott Laboratories, Columbus, OH) was added in the CM group when enteral intake reached 100 mL/[kg·day]. After starting fortification, milk intake was increased daily by 10–20 mL/kg to a maximum of 160 mL/[kg·day]. Nutritional content of the study diet was described previously.^1^

NEC was defined as Bell stage II disease or greater. Abdominal radiographs were read by radiologists unaware of study group assignment, regardless of the suspicion of NEC. All suspected and actual cases were adjudicated by a panel of neonatologists, also unaware of the study group assignment, before confirming the clinical diagnosis.^23^ After the study, all cases of NEC were reviewed in a blinded manner by a panel of eight of the study investigators. The original diagnosis of NEC was used for this current subgroup analysis.

Daily body weight and weekly recumbent length and head circumference were recorded. BPD was defined as use of supplemental oxygen at 36 weeks postmenstrual age (PMA); late-onset sepsis as clinical signs and symptoms consistent with sepsis occurring more than 5 days after birth with the isolation of a causative organism from a blood culture^24^ (on at least two occasions for coagulase-negative staphylococcus).

### Current trial subgroup reanalysis

Subgroup analysis was performed using data from those infants for whom MOM was 100% of base diet prefortification; so, the only difference between groups was whether CMDF or HMDF was used.

In the original trial, the HM40 and HM100 groups were merged, since the outcomes were the same. Merging was maintained for the current analysis after confirming no difference between these two HMDF groups in baseline characteristics or outcomes. This merging explains why the HMDF group had 82 subjects versus 32 in the CMDF group.

### Choice of outcomes for the current reanalysis

The main outcome for the current study was NEC (Bells' stage II or greater)—a predetermined outcome of the original trial. Our further main outcome, a severe morbidity index of NEC surgery or death, also derived from the findings of the original trial (Clinical Trials NCT00506584), in which NEC surgery was a major outcome, analyzed separately; and because of the high risk of immediate or later death in those requiring surgery, death is logically included since it has a censoring effect on the incidence of NEC surgery. When the original trial was analyzed jointly with the sister trial,^2^ there was a significant fourfold increased risk of all-cause death rate in the CM versus HM group (8% versus 2%).^8^ Also, analyzing NEC surgery or death as a combined index added power to this subgroup analysis given the smaller sample than in the original trial.

The secondary clinical outcomes included further morbidities where a higher incidence had been linked to CM exposure in other publications: BPD,^7,10–12^ requirement for mechanical ventilation,^7^ ROP,^7,9–11^ and sepsis.^5–8^ We also recorded growth.

### Statistical analysis

The baseline comparisons of categorical data used the chi-square test for homogeneity or Fisher's exact test^25^ for small cell sizes. Comparisons of baseline quantitative variables used the two-sample *t*-test. The unadjusted comparison of the rates of the different outcomes was based on the use of Fisher's exact test, while adjusted comparisons based on clinically relevant covariates utilized dichotomous logistic regression.^26^ Comparison of quantitative outcomes also used the Wilcoxon rank sum test.

## Results

**FIG. 1.**
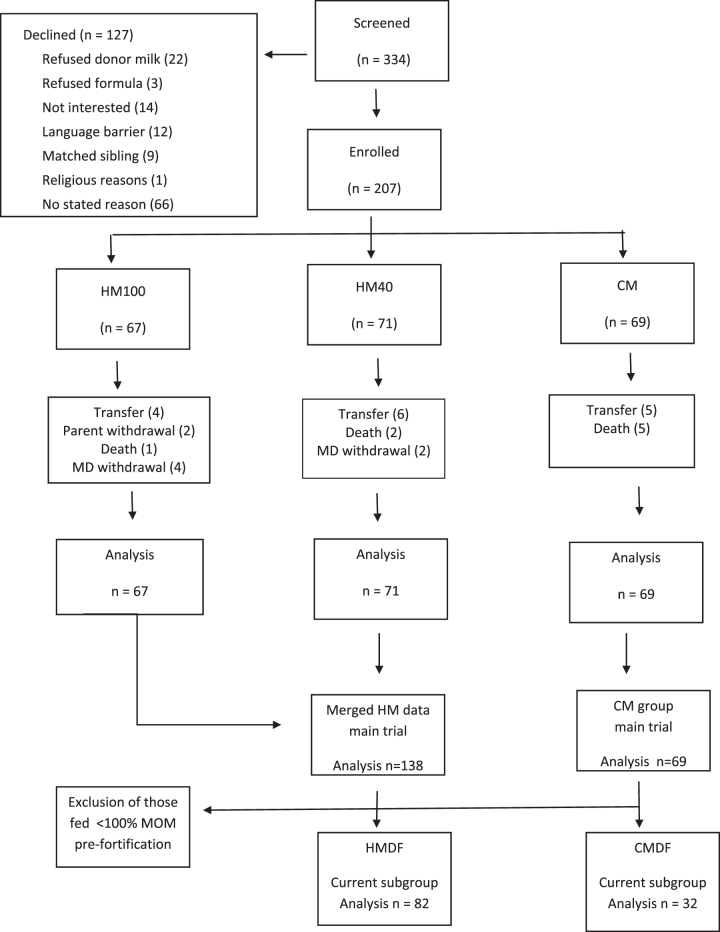
CONSORT diagram—distribution of study subjects. CM, group exposed to cow's milk-derived feeds (preterm formula or fortifier); CMDF, group fed cow's milk-derived fortifier; HM, group fed all human milk (HM100 or HM40); HM40, group fed human milk-derived fortifier with fortification starting at feed volume 40 mL/kg; HM100, group fed human milk-derived fortifier with fortification starting at feed volume 100 mL/kg; HMDF, group fed human milk-derived fortifier; MOM, mother's own milk.

Over 14 months, 334 infants were screened, and 207 enrolled in the original trial; and 114 were selected for this subgroup analysis (Fig. 1).

Baseline demographic factors were not significantly different between CMDF versus HMDF groups (Table 1), nor between this and the original study (not illustrated).

**Table 1.** Baseline Characteristics Comparing Infants Fed a Human Milk-Derived Fortifier Versus Cow's Milk-Derived Fortifier

Parameter	HMDF (*n* = 82)	CMDF (*n* = 32)	p^a^
Sex (female)	47/82 (57.3%)	15/32 (46.9%)	0.31
Race (black)	16/82 (19.5%)	3/32 (9.4%)	0.19
Antenatal steroids	67/82 (81.7%)	26/32 (81.2%)	0.95
APGAR <7	8/82 (9.8%)	6/32 (18.8%)	0.19
Gestation (weeks)	27.3 ± 2.2	27.1 ± 1.8	0.63
Birthweight (g)	937 ± 199	938 ± 190	0.98
SGA at birth	10/82 (12.2%)	3/32 (9.4%)	0.67

^a^ *t*-Test for quantitative variables and chi-square/Fisher's exact test for categorical variables.

CMDF, cow's milk-derived fortifier; HMDF, human milk-derived fortifier; SGA, small for gestational age.

The total hospitalization dose (intake) of MOM was equivalent in the two groups: 7.1 ± 3.8 L in the CMDF group and 6.8 ± 3.7 L in the HMDF group (*p* = 0.71). However, the proportion of MOM seen in this reanalysis (and in the original trial) was lower with HMDF versus CMDF (0.80 ± 0.09 versus 0.97 ± 0.01): entirely due to the liquid nature of the HMDF versus the powdered CMDF.

Fortifier was introduced earlier in the HMDF versus CMDF group: median 14.0 days versus 16.5 days (*p* = 0.03).

### Study outcomes

The incidence of Bell's stage II or greater NEC (Table 2)^23^ was significantly higher in the CMDF group 5/32 cases versus 3/82 cases—a relative risk (RR) of 4.2 (*p* = 0.038). After adjusting for race and Apgar score—the two baseline characteristics most different between groups (although not significantly so), the group difference was even more significant (*p* = 0.018).

**Table 2.** Primary and Secondary Outcomes Comparing Infants Fed a Human Milk-Derived Fortifier Versus Cow's-Milk Derived Fortifier

Parameter	HMDF (*n* = 82)	CMDF (*n* = 32)	p^a^
NEC (Bell Stage 2 or greater)	3/82 (3.7%)	5/32 (15.6%)	0.038
NEC surgery or death^b^	3/82 (3.7%)	6/32 (18.8%)	0.014
Surgical NEC^b^	1/82 (1.2%)	3/32 (9.4%)	0.066
Death^b^	3/82 (3.7%)	4/32 (12.5%)	0.096
BPD	24/82 (29.3%)	11/32 (34.4%)	0.60
Ventilator days	Median 9.5	Median 15.5	0.56
	IQR = 0.75, 41.25	IQR = 1, 50.25	
ROP (grade 3 or 4)	6/82 (7.3%)	2/32 (6.3%)	1.0

^a^ Chi-square/Fisher's exact test for categorical variables; for ventilator days, Wilcoxon's test.

^b^ Note that for the index “NEC surgery or death” there are three versus six cases in the HMDF and CMDF groups; this is one less in each group than the sum of NEC surgery and death when shown individually. This is because in each diet group, one case had *both* NEC surgery and death (not counted twice in the index).

BPD, bronchopulmonary dysplasia; CMDF, cow's milk-derived fortifier; HMDF, human milk-derived fortifier; ROP, retinopathy of prematurity.

For the severe morbidity index of NEC surgery or death (Table 2), 6/32 (18.8%) subjects in the CMDF group had a positive index compared to 3/82 (3.7%) in the HMDF group; an RR of 5.1 (*p* = 0.014). The number of subjects needed to harm was 7 (6.6). Thus, each seven infants treated with CMDF was associated with a death or case of NEC surgery.

### Exploratory analyses

In an exploratory analysis we took account of one protocol violator in the HMDF group (none in the CMDF group). This sole violator was the only subject that had NEC surgery in the HMDF group and hence had some influence. For the exploratory analysis, fortifier type and NEC, transferring the violator from the HMDF to the CMDF group changed the split of NEC cases to 6/32 (18.8) in the CMDF group versus 2/82 (2.4%) in the HMDF group; an RR of 7.8 (*p* = 0.008). For the severe morbidity index, the corresponding split became 7/32 (21.9%) in the CMDF group versus 2/82 (2.4%); RR = 9.1 (*p* = 0.002).

The incidence of NEC surgery and death in the two groups are shown separately (not combined as an index) as secondary outcomes in Table 2. All 7 deaths occurred between day 8 and 53 and were related to NEC or sepsis.

### Growth-related outcomes

Table 3 shows no difference in days to regain birth weight or in the rate of weight gain or length gain between the CMDF and HMDF groups. However, head circumference gain, reflecting brain growth, was 13% higher in the HMDF group; median 0.78 cm/week compared to 0.68 cm/week in the CMDF group (*p* = 0.04). None of the other outcomes was different between groups.

**Table 3.** Growth Characteristics of Infants Fed a Human Milk- Derived Fortifier Versus Cow's Milk-Derived Fortifier

Parameter	HMDF (*n* = 82)	CMDF (*n* = 32)	p^a^
Days to regain birthweight	Median = 6.5 (IQR = 3, 10)	Median = 6.0 (IQR = 2, 9)	0.24
Weight (g/[kg·day])	Median = 14.5 (IQR = 13.0, 15.9)	Median = 14.6 (IQR = 12.4, 16.2)	0.90
Length (cm/week)	Median = 0.90 (IQR = 0.68, 1.08)	Median = 0.88 (IQR = 0.63, 1.14)	0.58
HC (cm/week)	Median = 0.78 (IQR = 0.63, 0.88)	Median = 0.68 (IQR = 0.59, 0.80	0.04

^a^ Wilcoxon test.

CMDF, cow's milk-derived fortifier; HD, head circumference; HMDF, human milk-derived fortifier; IOR, interquartile range.

## Discussion

A rapidly emerging target in neonatal care, backed by official guidelines,^21,22^ is to feed small preterm infants a HM diet based on MOM and, if required, DM. Current common practice is to fortify these with a CMDF. Despite the increasing prevalence of this practice, safety aspects in comparison with HMDF have been minimally studied. Thus, while the use of CM-based products is linked to increased risk of a major morbidity,^1–20^ remarkably few data apply where fortifiers are used as the sole source of CM.

The development of HMDF^1,2^ has created the opportunity for randomized and nonrandomized controlled studies that could compare any morbidity associated with CMDF (when used as the sole source of CM) against HMDF as a non-CM-based comparison group; and where both groups received a base diet of HM. Despite the high intake of MOM, CMDF was still associated with a more than fourfold increased RR of NEC and over fivefold increased risk of severe morbidity comprising NEC surgery or death. While death itself is a major adverse outcome, it is important to include this in studies of NEC because of the censoring effect caused by death of babies who would otherwise be at higher risk of NEC had they survived. NEC cases requiring surgery have the worst prognosis for postneonatal complications, later death and adverse neurodevelopmental outcome. Thus, strategies to reduce NEC have major importance for the survival and quality of life of VLBW infants.

Two further studies add weight to our findings. The recently published OptiMoM trial^9^ in VLBW infants had an appropriate design to address safety of CMDF with modern practice. With a base diet of 100% HM (MOM and when required, DM), subjects were randomized to CMDF or HMDF. Severe ROP, was significantly more common in the CMDF group, in concordance with other studies on CM versus HM.^7,9–11^ There was a trend toward more late onset sepsis in the CMDF group (80% increase *p* = 0.07). These authors predefined a dichotomous morbidity index of death, NEC, ROP, BPD, or sepsis and following a recent published corrigendum the data showed a trend to a higher risk of a positive morbidity index in the CMDF group (40% higher, *p* = 0.07).^27^ Thus, as in our own study, there were safety concerns relating to CMDF.

Second, the RCT of Lucas et al.^28^ involved 276 preterm infants fed a base diet of MOM with PTF if required. Although the base diet was not 100% MOM, the study had the advantage of being conducted when CMDFs were introduced, ethically permitting randomisation to CMDF or to no fortification. The trial showed that the addition of CMDF to breast milk as the sole intervention more than doubled the combined incidence of confirmed NEC or proven sepsis compared to the no-treatment limb.

While others have shown CM exposure increased risk of ROP and sepsis, we did not show that here.^5–12^ This may be due to the high use of MOM in both limbs (100% of base diet). Also, RR of these outcomes with CM, as shown in other studies, is not as high as for NEC, necessitating a larger sample.

Those fed with CMDF and HMDF had similar growth in weight and length; and the median weight gain was 14.5 and 14.6 g/[kg·day], respectively. These latter figures reflect lower rates of growth than currently targeted, but are nevertheless close to the mean weight gain of 14.6 g/[kg·day] from 1 week postnatally to 34 weeks PMA in infants born at 27.6 weeks gestation in the Preterm Multi-Center Growth Study.^29^ However, the CMDF group had a moderate 13% lower rate in head circumference gain, and hence reduced brain growth, compared with the HMDF group. This requires investigation in further studies. One hypothesis explains that this is the high concentration of HM fat in the HMDF that could supply HM fat globule membrane^30^ or component lipids such as sphingomyelin that could promote white matter formation and cognitive development.^31^

We speculate the adverse effects of CMDF, despite a 100% HM base diet, could relate to the unexpectedly high dose of CM protein derived from fortifier, around 50% of protein intake. A current hypothesis is that CM may cause dysbiosis of the microbiome that precedes NEC and may be part of the causal sequence,^32^ although there is a paucity of evidence on whether there is any differential impact of PTF versus CMDF on the microbiome. A further potential factor is that when CM is directly added to MOM, it may diminish its protective properties.^33^

### Limitations

Our subgroup analysis could have created imbalances between the groups. However, the groups were well balanced for demographic factors and adjusting for race and Apgar score actually increased the significance of the association of CMDF with NEC. The greater morbidity in the CMDF group could potentially reflect an underlying higher risk population rather than a causal adverse effect of CMDF. We suggest the evidence points strongly against that. Our hypothesis was an a priori one driven by key outcomes in the original trial. Baseline risk factors were well balanced between groups. A large body of evidence links CM exposure to risk of NEC; and the effect size was large.

The CMDF subgroup had only 32 subjects who fulfilled our selection criteria. While this might have raised concern over the potential for type II error, this was not relevant here since the hypothesized dietary effect was significant; and a type I error is controlled by the significance level used (5%).

As noted, our study findings apply to the situation where the base diet is all MOM as data from DM studies were not specifically analyzed.

Our original trial population, although comprising very small infants, had a higher NEC rate than commonly seen today. Nevertheless, our findings that NEC was not reduced with sole use of CMDF as the CM source and that NEC rates were higher with CMDF versus HMDF remain the key observations in our study.

Finally, our findings apply to intact protein fortifiers in widespread international use. We did not consider here partially or extensively hydrolyzed fortifiers, now used in the United States. Such fortifiers have been compared with each other with some differences,^34–37^ but not compared to HMDF for the broad range of morbidities differentially affected by CM versus HM exposure.

## Conclusion

In a subgroup analysis of a RCT, we have used the opportunity to study the safety of CMDF compared to HMDF in VLBW premature babies fed a base diet of all MOM. Our findings showed adverse effects of using CMDF compared to HMDF with a 4.2-fold increased risk of NEC and a 5.1-fold increased risk of NEC surgery or death. Thus, those fed a HMDF were significantly advantaged in terms of a reduced incidence of morbidity. These data may help to underpin future strategies for quality improvement in neonatal nutrition.

## References

[1] Sullivan S, Schanler R, Kim J, et al. An exclusively human milk-based diet is associated with a lower rate of necrotizing enterocolitis than a diet of human milk and bovine milk-based products. J Pediatr 2010;156:562–567.e1 20036378 10.1016/j.jpeds.2009.10.040

[2] Cristofalo E, Schanler R, Blanco C, et al. Randomized trial of exclusive human milk versus preterm formula diets in extremely premature infants. J Pediatr 2013;163:1592–1595.e1 23968744 10.1016/j.jpeds.2013.07.011

[3] Quigley M, Embleton N, McGuire W Formula versus donor breast milk for feeding preterm or low birth weight infants. Cochrane Database Syst Rev 2018;6:CD002971 29926476 10.1002/14651858.CD002971.pub4 PMC6513381

[4] Lucas A, Cole T Breast milk and neonatal necrotising enterocolitis. Lancet 1990;336:1519–1523 1979363 10.1016/0140-6736(90)93304-8

[5] Narayanan I, Prakash K, Murthy NS, et al. Randomised controlled trial of effect of raw and holder pasteurised human milk and of formula supplements on incidence of neonatal infection. Lancet 1984;ii:1111-3 10.1016/s0140-6736(84)91554-x 6150180

[6] de Silva A Does human milk reduce infection rates in preterm infants? A systematic review. Arch Dis Child Fetal Neonatal Ed 2004;89:F509–F513 15499143 10.1136/adc.2003.045682 PMC1721772

[7] Hair A, Rechtman D, Lee M, et al. Beyond necrotizing enterocolitis: other clinical advantages of an exclusive human milk diet. Breastfeeding Med 2018;13:408–411 10.1089/bfm.2017.0192 PMC6065515 29877722

[8] Abrams S, Schanler R, Lee M, et al. Greater mortality and morbidity in extremely preterm infants fed a diet containing cow milk protein products. Breastfeed Med 2014;9:281–285 24867268 10.1089/bfm.2014.0024 PMC4074755

[9] O'Connor D, Kiss A, Tomlinson C, et al. Nutrient enrichment of human milk with human and bovine milk–based fortifiers for infants born weighing <1250 g: A randomized clinical trial. Am J Clin Nutr 2018;108:108–116 29878061 10.1093/ajcn/nqy067

[10] Assad M, Elliott M, Abraham J Decreased cost and improved feeding tolerance in VLBW infants fed an exclusive human milk diet. J Perinatol 2015;36:216–220 26562370 10.1038/jp.2015.168

[11] Manzoni P, Stolfi I, Pedicino R, et al. Human milk feeding prevents retinopathy of prematurity (ROP) in preterm VLBW neonates. Early Hum Dev 2013;89:S64–S68 23809355 10.1016/S0378-3782(13)70019-7

[12] Colacci M, Murthy K, DeRegnier R, et al. Growth and development in extremely low birth weight infants after the introduction of exclusive human milk feedings. Am J Perinatol 2017;34:130–137 27322667 10.1055/s-0036-1584520

[13] Anderson J, Johnstone B, Remley D Breast-feeding and cognitive development: A meta-analysis. Am J Clin Nutr 1999;70:525–535 10500022 10.1093/ajcn/70.4.525

[14] Lucas A, Morley R, Cole T, et al. Breast milk and subsequent intelligence quotient in children born preterm. Int J Gynecol Obst 1992;39:164–164 10.1016/0140-6736(92)91329-7 1346280

[15] Singhal A, Cole T, Lucas A Early nutrition in preterm infants and later blood pressure: Two cohorts after randomised trials. Lancet 2001;357:413–419 11273059 10.1016/S0140-6736(00)04004-6

[16] Singhal A, Fewtrell M, Cole T, Lucas A Low nutrient intake and early growth for later insulin resistance in adolescents born preterm. Lancet 2003;361:1089–1097 12672313 10.1016/S0140-6736(03)12895-4

[17] Singhal A, Cole T, Fewtrell M, et al. Breastmilk feeding and lipoprotein profile in adolescents born preterm: Follow-up of a prospective randomised study. Lancet 2004;363:1571–1578 15145629 10.1016/S0140-6736(04)16198-9

[18] Fewtrell M, Williams J, Singhal A, et al. Early diet and peak bone mass: 20 year follow-up of a randomized trial of early diet in infants born preterm. Bone 2009;45:142–149 19306955 10.1016/j.bone.2009.03.657

[19] Lucas A, Brooke O, Morley R, et al. Early diet of preterm infants and development of allergic or atopic disease: Randomised prospective study. BMJ 1990;300:837–840 2186825 10.1136/bmj.300.6728.837 PMC1662577

[20] Lewandowski A, Lamata P, Francis J, et al. Breast milk consumption in preterm neonates and cardiac shape in adulthood. Pediatrics 2016;138:e20160050–e20160050 27302980 10.1542/peds.2016-0050 PMC6198929

[21] AAP Section on Breastfeeding. Breastfeeding and the use of human milk. Pediatrics 2012;129:e827–e841 22371471 10.1542/peds.2011-3552

[22] ESPGHAN Committee on Nutrition, Arslanoglu S, Corpeleijn W, Moro G, et al. Donor human milk for preterm infants: Current evidence and research directions. J Pediatr Gastroenterol Nutr 2013;57:535–542 24084373 10.1097/MPG.0b013e3182a3af0a

[23] Walsh M, Kliegman R Necrotizing enterocolitis: Treatment based on staging criteria. Pediatr Clin North Am 1986;33:179–201 3081865 10.1016/S0031-3955(16)34975-6 PMC7131118

[24] Schanler RJ, Lau C, Hurst NM, et al. Randomized trial of donor human milk versus preterm formula as substitutes for mothers' own milk in the feeding of extremely premature infants. Pediatrics 2005;116:400-406 16061595 10.1542/peds.2004-1974

[25] Fisher RA On the interpretation of χ2 from contingency tables, and the calculation of P. J R Stat Soc 1922;85:87–94

[26] Walker SH, Duncan DB Estimation of the probability of an event as a function of several independent variables. Biometrika 1967;54 :167–178 6049533

[27] Corrigendum for O'Connor et al. (2019). Nutrient enrichment of human milk with human and bovine milk–based fortifiers for infants born weighing <1250 g: A randomized clinical trial. Am J Clin Nutr 2018;108:108–116. Am J Clin Nutr 2019;110:529–529 29878061 10.1093/ajcn/nqy067

[28] Lucas A, Fewtrell M, Morley R, et al. Randomized outcome trial of human milk fortification and developmental outcome in preterm infants. Am J Clin Nutr 1996;64:142–151 8694013 10.1093/ajcn/64.2.142

[29] Fenton T, Nasser R, Eliasziw M, et al. Validating the weight gain of preterm infants between the reference growth curve of the fetus and the term infant. BMC Pediatr 2013;13:92 23758808 10.1186/1471-2431-13-92 PMC3700759

[30] Timby N, Domellöf M, Lönnerdal B, et al. Supplementation of infant formula with bovine milk fat globule membranes. Adv Nutr 2017;8:351–355 28298277 10.3945/an.116.014142 PMC5347108

[31] Tanaka K, Hosozawa M, Kudo N, et al. The pilot study: Sphingomyelin-fortified milk has a positive association with the neurobehavioural development of very low birth weight infants during infancy, randomized control trial. Brain Dev 2013;35:45–52 22633446 10.1016/j.braindev.2012.03.004

[32] Pammi M, Cope J, Tarr PI, et al. Intestinal dysbiosis in preterm infants preceding necrotizing enterocolitis: A systematic review and meta-analysis. Microbiome 27;5:31 28274256 10.1186/s40168-017-0248-8 PMC5343300

[33] Chan G, Lee M, Rechtman D Effects of a human milk-derived human milk fortifier on the antibacterial actions of human milk. Breastfeed Med 2007;2:205–208 18081457 10.1089/bfm.2007.0015

[34] Kim J, Chan G, Schanler R, et al. Growth and tolerance of preterm infants fed a new extensively hydrolyzed liquid human milk fortifier. J Pediatr Gastroenterol Nutr 2015;61:665–671 26488118 10.1097/MPG.0000000000001010 PMC4645956

[35] Monga R, Sampath V, Ehrhart B, et al. Prospective comparison of enfamil and similac liquid human milk fortifier on clinical outcomes in premature infants. Am J Perinatol 2017;34:1411–1416 28637062 10.1055/s-0037-1603940

[36] Lainwala S, Kosyakova N, Spizzoucco A, et al. Clinical and nutritional outcomes of two liquid human milk fortifiers for premature infants. J Neonatal Perinatal Med 2017;10:393–401 29286933 10.3233/NPM-16164

[37] Ng DHC, Klassen JRL, Embleton ND, et al. Protein hydrolysate versus tandard formula for preterminfants. Cochrane Database Syst Rev 2019:CD012412 31339557 10.1002/14651858.CD012412.pub3 PMC6653062

